# Percutaneous rendezvous delivery of an intraductal metal stent for a refractory biliary stricture after liver transplantation

**DOI:** 10.1055/a-2965-6026

**Published:** 2026-10-08

**Authors:** Eisuke Ozawa, Kosuke Takahashi, Mizuki Kitagawa, Masashi Shibata, Hideki Ishimaru, Susumu Eguchi, Hisamitsu Miyaaki

**Affiliations:** 1 Department of Gastroenterology and Hepatology 200674 Nagasaki University Graduate School of Biomedical SciencesNagasakiJapan; 2 Department of Radiology 200674 Nagasaki University Graduate School of Biomedical SciencesNagasakiJapan; 3 Department of Surgery, Division of Abdominal and Transplant/Regenerative Surgery 88380 Nagasaki University Graduate School of Biomedical SciencesNagasakiJapan

A 71-year-old woman underwent living-donor liver transplantation (LDLT) with a left-lobe graft and duct-to-duct reconstruction. Eighteen days later, computed tomography showed intrahepatic biliary dilation from an anastomotic stricture ( Fig. 1a ).

**Fig. 1:**
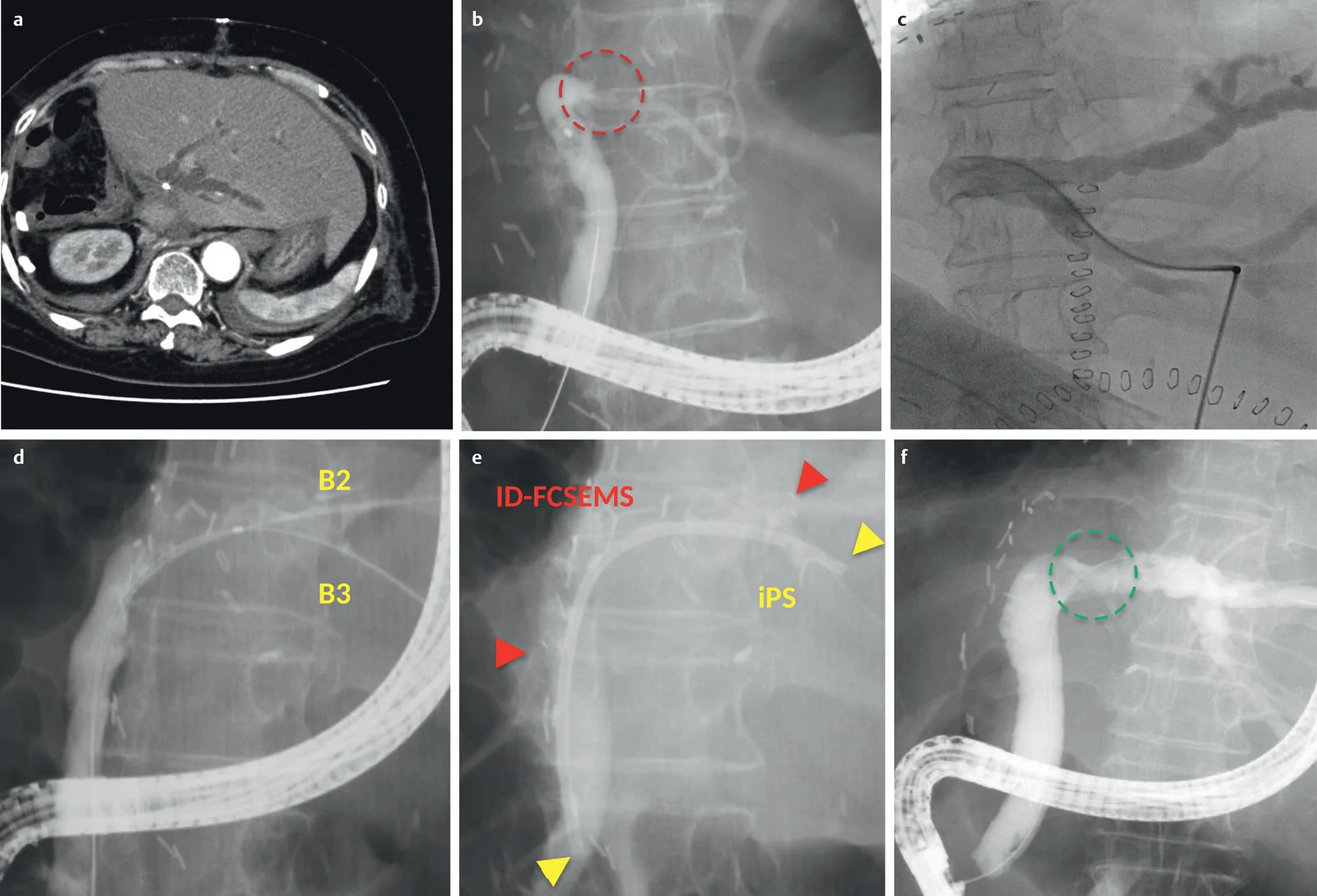
( **a** ) Contrast-enhanced computed tomography after living-donor liver transplantation with a left-lobe graft: the intrahepatic bile ducts are dilated. ( **b** ) Attempted endoscopic biliary drainage—the papilla was easily cannulated, but at two attempts the anastomosis (a red dashed circle) was too tight to opacify the graft ducts and no guidewire could be advanced into them. ( **c** ) Percutaneous transhepatic cholangiodrainage through the B3 duct. ( **d** ) The technical step of this report: after the rendezvous, an uneven double-lumen cannula railed over the captured wire holds it in B3 through the tip lumen while a second guidewire is steered into B2 through the side lumen, so both graft ducts are wired at once. ( **e** ) Combined intraductal stenting: red arrowheads mark the two ends of the intraductal fully covered self-expandable metal stent (ID-FCSEMS; BONASTENT M-Intraductal) in B2 and yellow arrowheads at the two ends of the inside plastic stent (iPS) in B3, both stents lying entirely above the papilla. ( **f** ) Cholangiography at stent removal, 16 weeks after placement: the anastomosis (a green dashed circle) is now widely patent.

At endoscopic retrograde cholangiopancreatography, cannulation was straightforward, but two attempts failed to cross the anastomosis ( Fig. 1b ). Percutaneous transhepatic cholangiodrainage (PTCD) was performed through the B3 bile duct ( Fig. 1c ). Retrograde access was never achievable, so each recurrence was managed by repeating the rendezvous and exchanging plastic stents.

Judging the stricture refractory, we used the rendezvous to deliver an intraductal fully covered self-expandable metal stent (ID-FCSEMS; Video 1 ). A guidewire was advanced through the PTCD tract, across the stricture and out of the papilla, where a snare drew it into the working channel. An uneven double-lumen cannula (PIOLAX, Tokyo, Japan) was railed over it: the 0.025-inch tip lumen held the wire in B3 while a second guidewire entered B2 through the 0.035-inch lumen 3 cm proximal ( Fig. 1d ).

**Video 1** Percutaneous transhepatic rendezvous delivers an intraductal fully covered metal stent across a refractory anastomotic biliary stricture after living-donor liver transplantation, with bilateral guidewire access from one wire.


After balloon dilation, a 7-Fr plastic stent was placed into B3 and a 10-mm × 6-cm ID-FCSEMS into B2 ( Fig. 1e ). Sixteen weeks later, the ID-FCSEMS was removed endoscopically; the anastomosis was widely patent ( Fig. 1f ). No adverse events occurred; the stricture has not recurred over 5 years.

Rendezvous restores access 1​ ​2​ and the ID-FCSEMS resolves refractory strictures; 3​ ​4​ the guidewire from the uneven cannula entered a second duct during endoscopic ultrasound-guided drainage 5​ ( Fig. 2a, b ). We found no report combining them. Intraductal placement spares the sphincter and avoids duodenobiliary reflux ( Fig. 2c ). Because the wire is held outside the body, devices push against a fixed rail and cross strictures that would otherwise deflect them. The percutaneous route is also sterile—useful under immunosuppression. In duct-to-duct LDLT, rendezvous series end with plastic stents; 1​ ​2​ here, the rendezvous itself delivered the definitive stent.

**Fig. 2:**
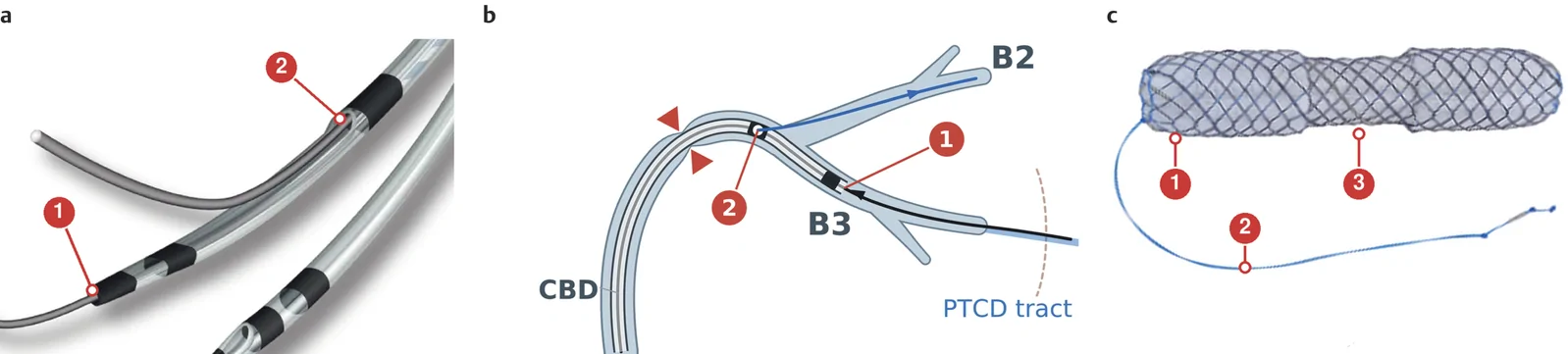
The two-guidewire strategy and the devices it uses. ( **a** ) Uneven double-lumen cannula (PIOLAX, Tokyo, Japan): 1. the 0.025-inch lumen opens at the distal tip; 2. the 0.035-inch lumen opens through a side orifice 3 cm proximal to it. ( **b** ) Schematic of how the two lumens are used, drawn from this patient’s own cholangiogram. The rendezvous wire (black), won through the percutaneous transhepatic tract in B3 and captured at the papilla, stays in the tip lumen (1), which rails the cannula (grey) across the anastomotic stricture (red arrowheads mark the narrowed segment) into B3; the side orifice (2) then delivers a second guidewire (blue) into B2. CBD, common bile duct. ( **c** ) An intraductal fully covered self-expandable metal stent (BONASTENT M-Intraductal, Standard Sci-Tech, Seoul, Korea): 1. a fully covered braided mesh, 10 mm × 6 cm, short enough to lie entirely above the papilla; 2. the long lasso is grasped at removal; 3. the constricted central waist straddles the stricture and anchors the stent. Source: Panels **a** and **c** are device product images, reproduced with the written permission of PIOLAX Medical Devices, Inc. and Medicos Hirata Inc. respectively; panel **b** is an original drawing [rerif].

Endoscopy_UCTN_Code_TTT_1AR_2AZ

## Declaration of GenAI Use

A large language model (Anthropic Claude) was used as a drafting and illustration aid: it helped edit the English text, assemble and de-identify the video from the authors’ own footage, and render the schematic panel of Fig. 2b from a drawing made by the authors. All clinical contents, images and footage originate from the authors, who verified every statement and figure and take full responsibility for the manuscript.
